# Spiegelzymes® Mirror-Image Hammerhead Ribozymes and Mirror-Image DNAzymes, an Alternative to siRNAs and microRNAs to Cleave mRNAs *In Vivo*?

**DOI:** 10.1371/journal.pone.0086673

**Published:** 2014-01-29

**Authors:** Eliza Wyszko, Florian Mueller, Marta Gabryelska, Angelika Bondzio, Mariusz Popenda, Jan Barciszewski, Volker A. Erdmann

**Affiliations:** 1 Institute of Bioorganic Chemistry of the Polish Academy of Sciences, Poznan, Poland; 2 Pentafolium-Soft, Rosengarten, Germany; 3 Institute for Biochemistry, Veterinary Medicine, Free University of Berlin, Berlin, Germany; 4 Institute for Chemistry and Biochemistry, Free University of Berlin, Berlin, Germany; 5 Erdmann Technologies GmbH, Berlin, Germany; University of Helsinki, Finland

## Abstract

With the discovery of small non-coding RNA (ncRNA) molecules as regulators for cellular processes, it became intriguing to develop technologies by which these regulators can be applied in molecular biology and molecular medicine. The application of ncRNAs has significantly increased our knowledge about the regulation and functions of a number of proteins in the cell. It is surprising that similar successes in applying these small ncRNAs in biotechnology and molecular medicine have so far been very limited. The reasons for these observations may lie in the high complexity in which these RNA regulators function in the cells and problems with their delivery, stability and specificity. Recently, we have described mirror-image hammerhead ribozymes and DNAzymes (Spiegelzymes®) which can sequence-specifically hydrolyse mirror-image nucleic acids, such as our mirror-image aptamers (Spiegelmers) discovered earlier. In this paper, we show for the first time that Spiegelzymes are capable of recognising complementary enantiomeric substrates (D-nucleic acids), and that they efficiently hydrolyse them at submillimolar magnesium concentrations and at physiologically relevant conditions. The Spiegelzymes are very stable in human sera, and do not require any protein factors for their function. They have the additional advantages of being non-toxic and non-immunogenic. The Spiegelzymes can be used for RNA silencing and also as therapeutic and diagnostic tools in medicine. We performed extensive three-dimensional molecular modelling experiments with mirror-image hammerhead ribozymes and DNAzymes interacting with D-RNA targets. We propose a model in which L/D-double helix structures can be formed by natural Watson-Crick base pairs, but where the nucleosides of one of the two strands will occur in an anticlinal conformation. Interestingly enough, the duplexes (L-RNA/D-RNA and L-DNA/D-RNA) in these models can show either right- or left-handedness. This is a very new observation, suggesting that molecular symmetry of enantiomeric nucleic acids is broken down.

## Introduction

A fundamental property of the biological proteins and nucleic acid macromolecules is their homochirality, i.e. that they are composed of only one optically active isomer of amino acids or nucleotides, respectively. Native proteins are composed exclusively of L-amino acids, while natural nucleic acids contain D-sugar residues. However, some D-amino acids and short D-oligopeptides are present in the cell walls of some bacteria [1] and the D-oligopeptides are made by non-ribosomal processes. Although the origin of the homochirality of biological macromolecules is not fully understood, it is assumed to be essential to ensure the high specificity and fidelity of recognition between them. Mirror-image ribonucleic acids consisting of L-ribose instead of D-ribose are resistant to hydrolysis by cellular nucleases [2]–[4]. Thus, L-nucleic acids should be suitable for the development of new tools for *in vivo* applications. Indeed mirror-image aptamers, called Spiegelmers, were developed more than 15 years ago [3]–[4]. The toxicological data for L-RNA aptamers intended for medical applications have not shown any adverse side-effects so far, even when applied in relatively high doses *in vivo* [5]. In addition, mirror-image RNAs did not induce any immunological responses, such as those observed upon application of some high molecular weight nucleic acids [5]–[7]. The great advantage of the application of nucleic acids as therapeutic agents is based on the recognition of relevant nucleic acid targets by complementary Watson-Crick base pairing. Such interactions, which include antisense oligonucleotides, siRNAs and catalytic RNAs (ribozymes), are widely used for controlling gene expression [8]. Interestingly, L-DNA was proposed for use in antisense technologies 20 years ago; however, no stable hybrids with D-RNA could convincingly be demonstrated [9]–[12].

In this paper, we show for the first time that mirror-image hammerhead ribozymes and DNAzymes (L-zymes) cleave sequence-specific complementary D-RNA, i.e. a target of different chirality. These mirror-image nucleic acid zymes, Spiegelzymes, have some features which could make them valuable for future therapeutic and diagnostic applications. The precise mechanism of action of the Spiegelzymes is not known and will require further investigation. However, what we are reporting in this communication are extensive three-dimensional molecular modelling studies, from which we currently favour a model in which the D-RNA targets are interacting with the Spiegelzymes by Watson-Crick interaction of the bases, and in which their sugar residues acquire a new type of structural arrangement**.**

## Materials and Methods

### RNA and DNA synthesis

All RNA and DNA oligonucleotides used in these studies were chemically synthesized at IBA (Germany) or ChemGenes (USA). The 14 nt synthetic D- and L-RNA oligomers, a fragment of GFP mRNA (252–265 nt), with the sequence 5′-C_1_UUCAAGUCCGCCA_14_-3′ are labelled with fluorescein at the 5′ end. In the target sequence, the hammerhead ribozyme hydrolyses the GUC sequence after the C and the DNAzyme hydrolyse the AGU sequence between the G and the U.

The HH ribozyme (D-RNA) and HH Spiegelzyme (L-RNA) were of 33 nt lengths with the sequence 5′-U_1_GGCGCUGAUGAGGCCGAAAGGCCGAAACUUGA_33_-3′. The 27-nucleotide-long sequences of the synthetic L- and D-DNAzymes were selected to be 5′-G_1_GCGGAGGCTAGCTACAACGATTGAAG_27_-3′.

### RNA hydrolysis with ribozymes in vitro

The activities of the HH Spiegelzyme and the HH ribozyme with fluorescein-labelled L- and D-RNA targets were measured in 10 µl reaction volumes containing 50 mM Tris-HCl buffer, pH 7.5, at 37°C. The target RNA and HH Spiegelzyme, or HH ribozyme, were denatured for 2 min at 73°C and cooled down in a heating block to 25°C at a rate of 1°C/min. Hydrolysis reactions were carried out at MgCl_2_ concentrations between 0 and 25 mM. Hydrolysis products were separated by 20% polyacrylamide gel electrophoresis (PAGE) in the presence of 7 M urea in 0.09 M Tris-borate buffer at pH 8.3. The reaction products were visualized with a Fuji Film FLA 5100 phosphoimager using the manufacturer's (Fuji) software.

### RNA hydrolysis with DNAzymes *in vitro*

An amount of 0.2 µM of fluorescein-labelled D- and L-RNA was incubated in 10 µl of a reaction mixture containing 50 mM Tris-HCl buffer, pH 7.5, and 0–25 mM MgCl_2_ at 37°C for 1–128 min or 5 h in the presence of 2–5 µM D- and L-DNAzyme. Prior to the reaction, the substrate (D- and L-RNA) and D- and L-DNAzyme were denatured, as described for the ribozyme experiments. The analysis of the reactions by PAGE was carried out as described above.

### Spiegelzyme activity in HeLa cells

The cells (2×10^5^) were plated in 500 µl of growth medium (DMEM, 10% FSC, antibiotics) to achieve 70–80% confluency 24 h before transfection in a 24-well format. The cells were transfected with plasmid pEGFP (1 µg) and HH Spiegelzyme or HH ribozyme in final concentrations of 25–300 nM, and 25–100 nM in the case of D- or L-DNAzymes using lipofectamine 2000. Changes in GFP fluorescence were monitored with a microscope (Leica) and plate reader.

### Spiegelzyme stability in COS-7 cells

The cells (2×10^5^) were plated in 500 µl of growth medium (DMEM, 10% FSC, antibiotics) to achieve 70–80% confluency. Transfection of Spiegelzyme (1 µg) with lipofectamine 2000 was performed according to the protocol from Ambion. Images were taken with a Leica 4000B instrument at 0, 24, 72, 96, and 120 h after transfection.

### Real-time quantitative PCR

Real-time qPCR was performed to assess the transcripts of GFP expression relative to Hprt and Actb. Each cDNA sample was analysed using a thermocycler Stratagene Mx3005P (Agilent Technologies). The 25 µl reaction mixture was prepared with a DyNAmo HS Sybr Green qPCR Kit (FINNZYMES) and included 1× MasterMix, 0.3× ROX reference dye, 0.3 µM of each primer, 1 µl of template cDNA, and water to a final volume of 25 µl. The PCR reaction conditions for all genes were as follows: initial denaturation (95°C, 10 min), a four step amplification programme repeated 40 times (95°C for 15 sec, 72°C for 30 sec), and a melting curve programme (95°C for 1 min, 55°C for 30 sec, 55–95°C with a heating rate of 0.1°C/sec, 95°C for 30 sec). Standard curves were generated by amplifying serials of five times dilutions of cDNA.

Results were reported as the relative expression to that of Actb and Hprt in the same samples. The quality of PCR products was checked by analysis of the melting curve. All experiments were repeated three times.

### Western blot

The cells (about 2.5×10^6^) were washed with PBS, resuspended in 10 mM Tris, pH 7.5, and centrifuged for 10 min at 4°C and 13.2×10^3^ rpm. Each sample was denatured by heating at 95°C for 10 min. The samples were subjected to electrophoresis in 0.1% SDS polyacrylamide gels, and separated proteins were transferred to a PVDF membrane (PerkinElmer) using Western Unit (BioRad) in Towbin buffer (25 mM Tris, pH 7.5, 190 mM glycine, 20% methanol). The membrane was blocked with 10% skimmed milk in 1×PBS/0.05%Tween20 at 4°C overnight, and afterwards, washed three times in 1×PBS/0.05%Tween20 at room temperature. The membrane was incubated with the antibodies for GAPDH (1∶500 dilution, Santa Cruz Biotechnology, GAPDH mouse monoclonal Ig 0411) and GFP (1∶500 dilution, Santa Cruz Biotechnology, GFP mouse monoclonal Ig A00185.01) for 2 h at room temperature. After washing three times in 1×PBS/0.05%Tween20, the membrane was treated with secondary Anti-Mouse Ig-Biotin antibody (1∶2 000 dilution, Sigma) for 2 h at room temperature. The membrane was washed three times and then incubated with Streptavidine Alkaline Phosphatase Conjugate for 15 min at room temperature. After washing, the membrane was developed using BCIP/NBT Liquid Substrate System (Sigma), and bands were quantified using the ImageQuant software from Molecular Dynamics. See also Table 1 for further details.

**Table 1:** Western Blot Table with Details for Actb, Hprt and Gfp Proteins analysed.

Name	Nucleotide sequence (5′-3′)	Tm (°C)	Fragment size (nt)	Characteristic
Actb	F-TCTGGCACCACACCTTCTAC	60	168	NM_001101.3
	R- GATAGCACAGCCTGGATAGC			
Hprt	F-CTGAGGATTTGGAAAGGGTG	60	155	NM_000194.2
	R-AATCCAGCAGGTCAGCAAAG			
Gfp	F-CCTGAAGTTCATCTGCACCA	60	120	PEGFP-N3-
	R-AAGTCGTGCTGCTTCATGTG			Clontech

## Results and Discussion

Recently, we have shown for the first time that L-hammerhead ribozymes and L-DNAzymes hydrolyse RNA of the same chirality with a very high efficiency [2]. Both Spiegelzymes and the substrate form a double-stranded, left-handed helix with regular Watson-Crick base pairs. In this study, we address for the first time the question, whether or not Spiegelzymes will be able to hydrolyse nucleic acid molecules of opposite chirality. Thus, we designed L-RNA hammerhead and L-DNA Spiegelzymes as site-specific tools to target naturally occurring D-RNA molecules.

**Figure 1:**
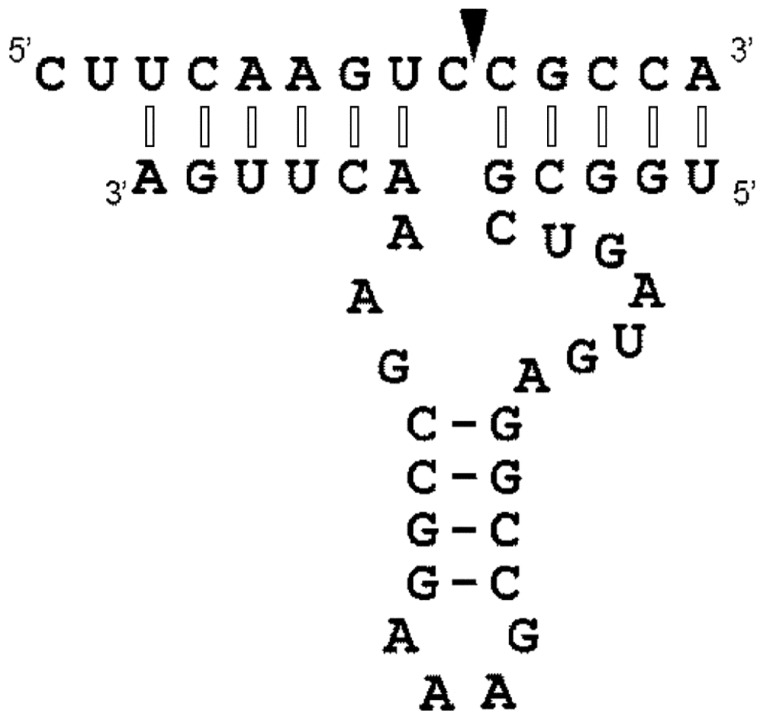
The secondary structural model of a hammerhead Spiegelzyme complexed with an D-RNA substrate containing GUC↓N, the required cleavage site for hammerhead ribozymes. The cleavage site is indicated with an arrow. Interactions between the hammerhead Spiegelzyme (L-RNA) and the D-RNA substrate of opposite chirality show modified Watson-Crick base pairs with nucleotides in anticlinal conformation (see Figs. 12–14) and are marked with open bars. The normal Watson-Crick bases in antiperiplanar conformation are indicated with closed bars.

Firstly, we chemically synthesized a 33-nucleotide-long mirror-image L-hammerhead ribozyme and a 14-nucleotide-long D-RNA substrate. The target sequence was selected in such a way that it corresponded to a part of a GFP mRNA, which included the required **GUC**↓N cleavage sequence for hammerhead ribozymes (Fig. 1).

**Figure 2:**
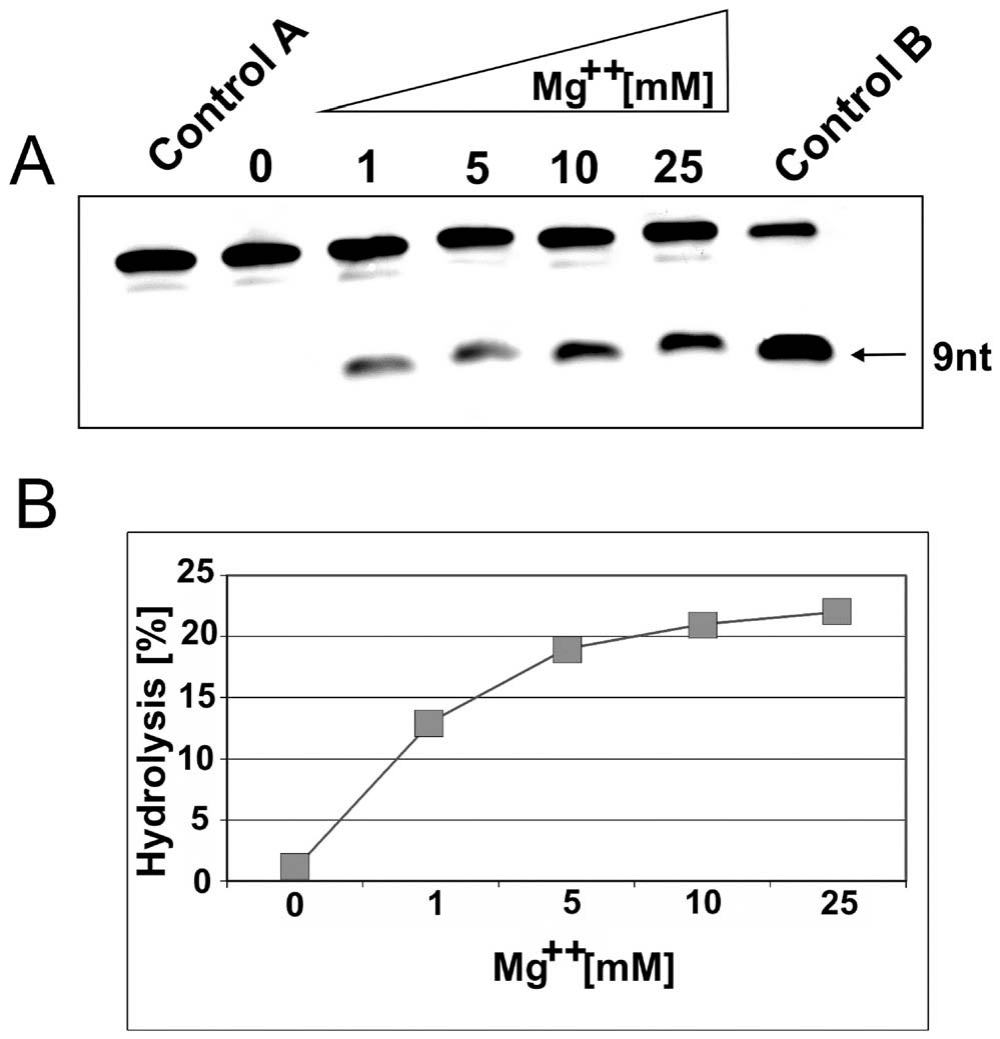
Magnesium dependence of D-RNA hydrolysis by hammerhead Spiegelzymes. **A.** D-RNA (0.2 µM) was incubated with 2 µM of the Spiegelzyme in 50 mM Tris-HCl, pH 7.5, buffer containing 1, 5, 10, and 25 mM MgCl_2_ for 1 h at 37°C. The reaction products were separated by 20% polyacrylamide gel electrophoresis with 7 M urea. Control reactions were carried out with target D-RNA alone in buffer (lane Control A) or with D-RNA hammerhead ribozyme in buffer with 10 mM Mg^2+^ (lane Control B). **B**. Hydrolytic activity in % of hammerhead Spiegelzyme with the D-RNA target at different magnesium concentrations.

At first, we looked for an Mg^++^ effect on the heterochiral complex formation and L-hammerhead ribozyme activity. As can be seen, without magnesium, the cleavage reaction does not take place (Fig. 2). Thus, the RNA hydrolysis reaction is clearly dependent on the magnesium ion and reached an optimal activity at 10 mM MgCl_2_.

**Figure 3:**
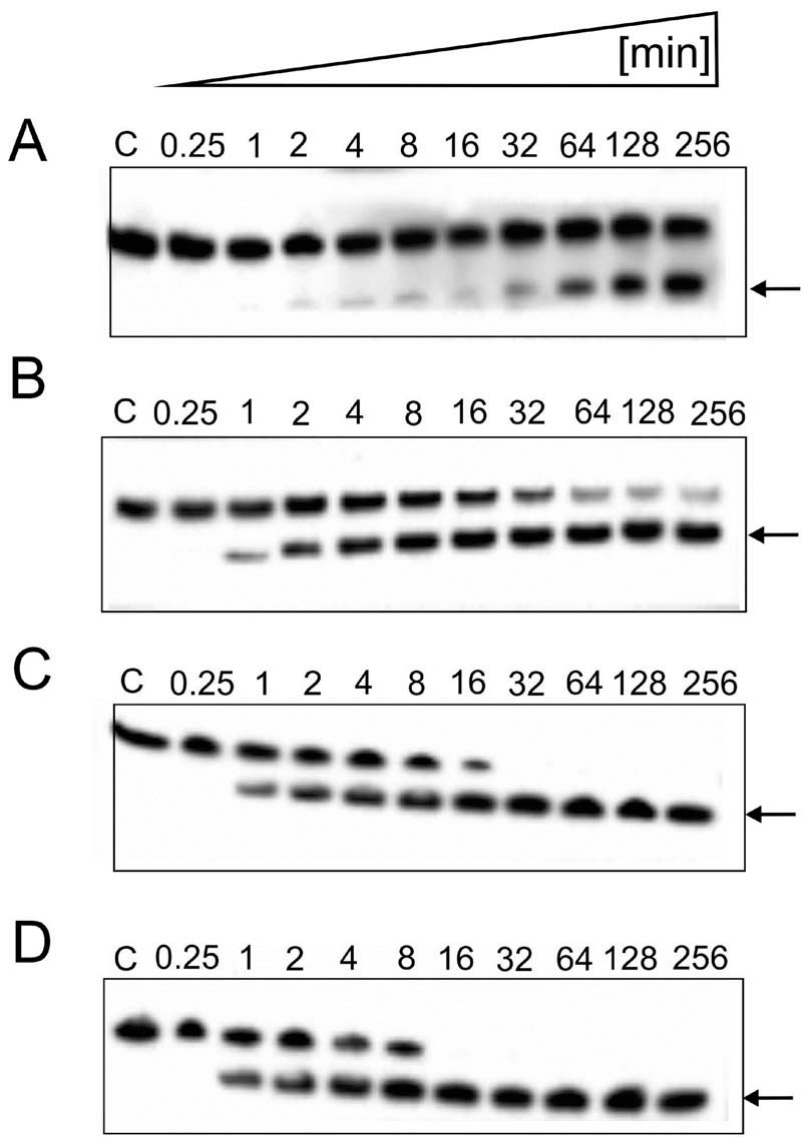
Time-dependent cleavage of the D-RNA substrates by the hammerhead Spiegelzyme. Reactions were carried out at 37°C in a 50 mM Tris-HCl, pH 7.5, buffer, containing 10 mM MgCl_2_. Reaction products were separated by a 20% polyacrylamide gel electrophoresis containing 7 M urea in 0.09 M Tris-borate buffer at pH 8.3. The fluorescence was analysed by a Fuji Film FLA 5100 phosphoimager. The hydrolysis of 0.2 µM fluorescein-labelled D-RNA with hammerhead Spiegelzyme to substrate ratios of 10∶1 (A) 25∶1 (B), 50∶1 (C), and 100∶1 (D) are shown in this figure. The arrows indicate the expected hydrolysis product of 9-nucleotide length.

Next, we checked a ratio of Spiegelzyme to substrate RNA. The data shown in Fig. 3 demonstrates that, under the conditions chosen, 100% hydrolysis will be achieved in 16 min at a 100-fold excess of the Spiegelzyme. Therefore, we decided to use longer incubation times and enzyme to substrate ratios of 10∶1 or 25∶1 for the following experiments.

**Figure 4:**
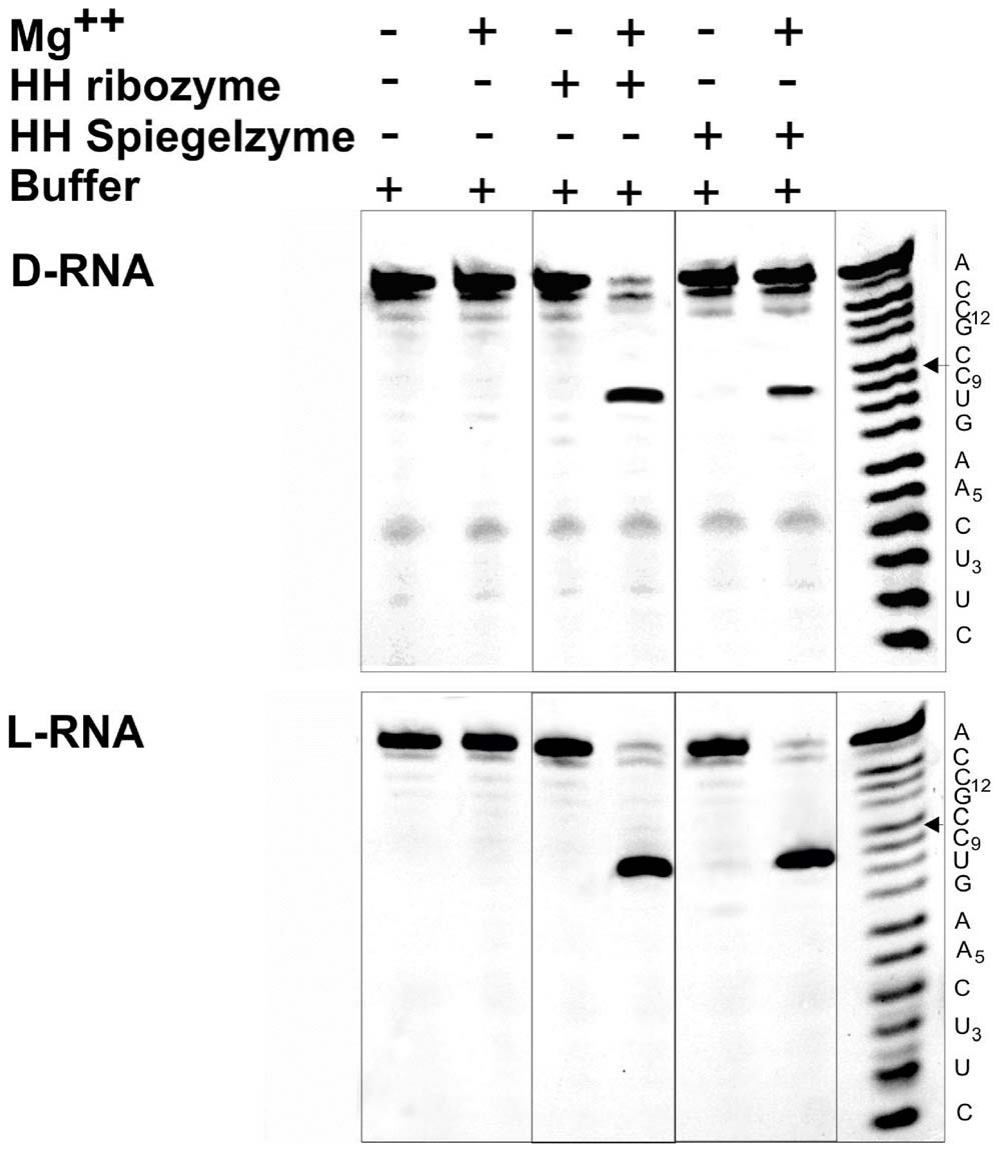
Hydrolysis of D-RNA and L-RNA substrates by hammerhead ribozymes and hammerhead Spiegelzymes. The substrates (0.2 µM) were incubated with 2 µM of hammerhead ribozymes or hammerhead Spiegelzymes in 50 mM Tris-HCl, pH 7.5, buffer, at 37°C for 2 h. 10 mM MgCl_2_ and hammerhead ribozymes or hammerhead Spiegelzymes were added as indicated. In the top panel, D-RNA substrates and in the bottom panel, L-RNA substrates were used. Hydrolysis products were separated on 20% polyacrylamide gel electrophoreses with 7 M urea. The ladder (right) was generated by alkaline hydrolysis.

Both hammerhead ribozyme and hammerhead Spiegelzyme cleave D- and L-RNA substrates with comparable activities, as shown in Fig. 4. These data confirmed our earlier observations that the ribozyme specificities in homochiral (D/D and L/L) complexes are preserved and occur at the predetermined **GUC**↓N cleavage site of the substrate [2]. Therefore, it is reasonable to conclude that homochiral (D/D and L/L) RNA complexes follow similar folding patterns. Our results shown in Fig. 4 are extremely interesting; we not only observed that the hammerhead Spiegelzyme is cleaving the D-RNA substrate, but that the GUC↓N cleavage site has also been conserved.

**Figure 5:**
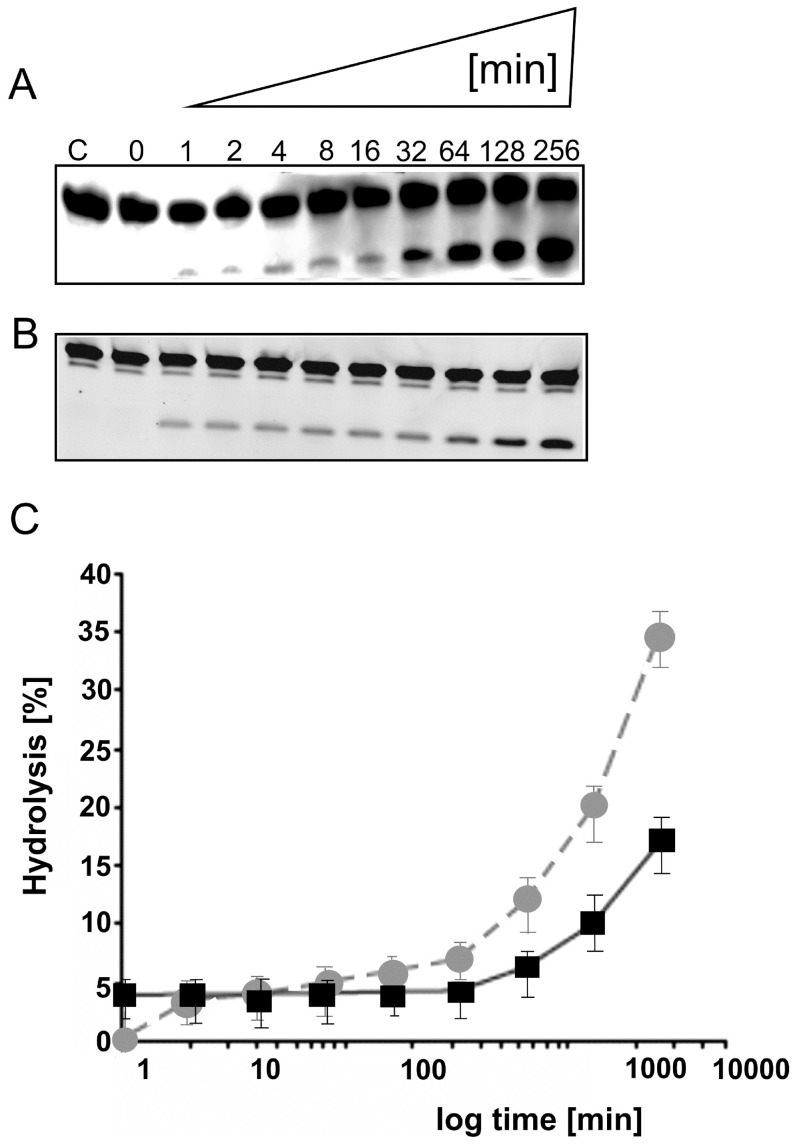
Time-dependent cleavage of the D-RNA target with the hammerhead Spiegelzyme (heterochiral complex). The incubation of 0.2 µM target fluorescein-labelled D-RNA, with 2 µM or 0.02 µM hammerhead Spiegelzyme for single **(A)** and multiple (**B**) turnover reactions. The reactions were carried out in 50 mM Tris-HCl, pH 7.5, buffer containing 10 mM MgCl_2_ at 37°C and different incubation times as indicated. Control reaction was carried out with target D-RNA alone in buffer for 256 min (lane C). Reaction products were separated by 20% polyacrylamide gel electrophoreses containing 7 M urea and analysed by a Fuji Film FLA 5100 phosphoimager**. (C)** A plot of single (squares, black solid line) and multiple (circle, grey broken line) turnover reactions. The time of reaction is shown in logarithmic scale.

The cleavage reaction of D-RNA at single turnover conditions in the presence of a 10-fold excess of hammerhead Spiegelzyme over the substrate showed a k_obs_ of 4.52×10^−2^ min^−1^, which is comparable with the k_obs_ (1.5×10^−2^ min^−1^) for the homochiral system (Fig. 5). This observation was surprising. It has so far been reported that only anti-parallel Watson-Crick type base pairing allows the formation of double-stranded nucleic acids of homochiral complexes of D-RNA target with D-RNA enzymes or their mirror images [2], [13].

**Figure 6:**
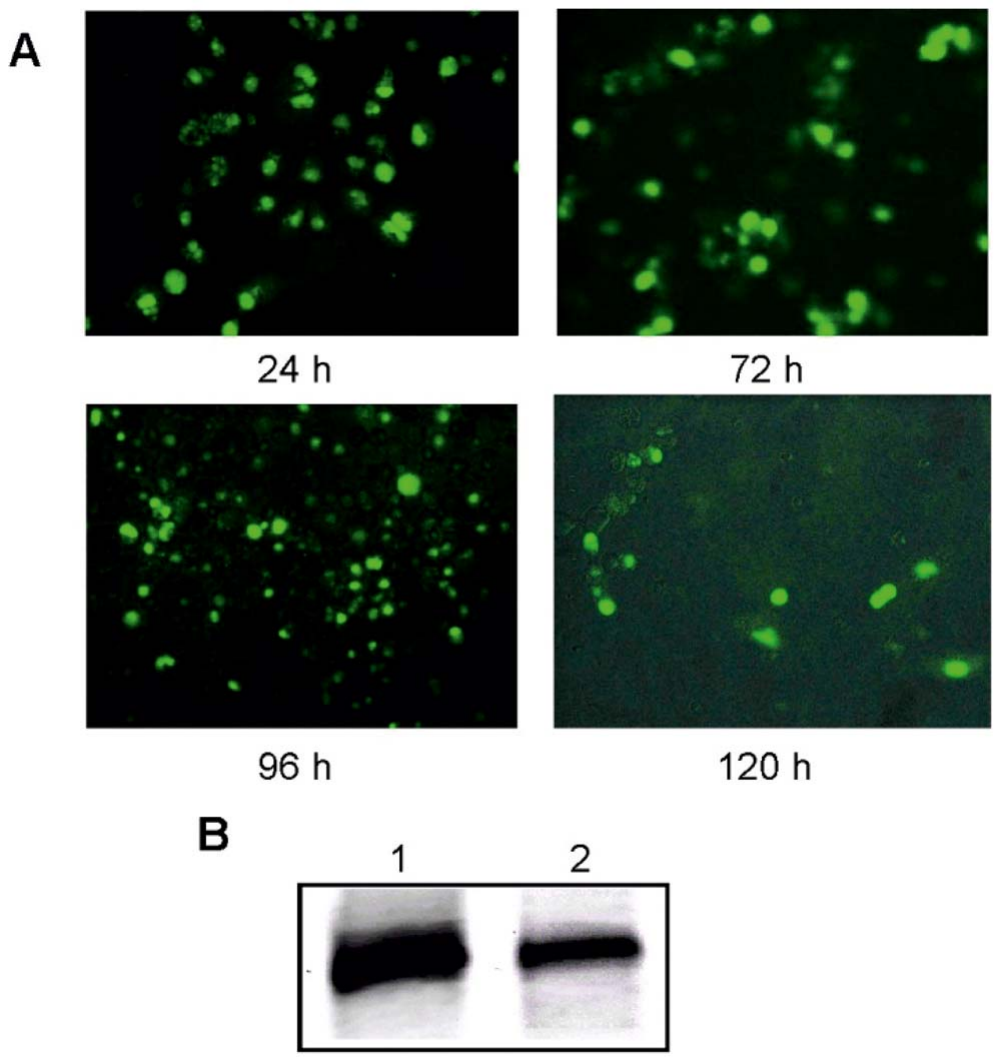
Hammerhead Spiegelzyme stability analysis in COS-7 cells. **A.** 5′-fluorescein-labelled Spiegelzyme (1 µg) was transfected into cells with Lipofectamine 2000. The microscope images were taken at 24, 72, 96, and 120 h after transfection. **B.** The RNA isolated from transfected cells (Trizol, Ambion) was analysed on 20% PAGE with 7 M urea. Lane 1 control hammerhead Spiegelzyme. Spiegelzyme after 120 h of incubation in COS-7 cells (lane 2).

**Figure 7:**
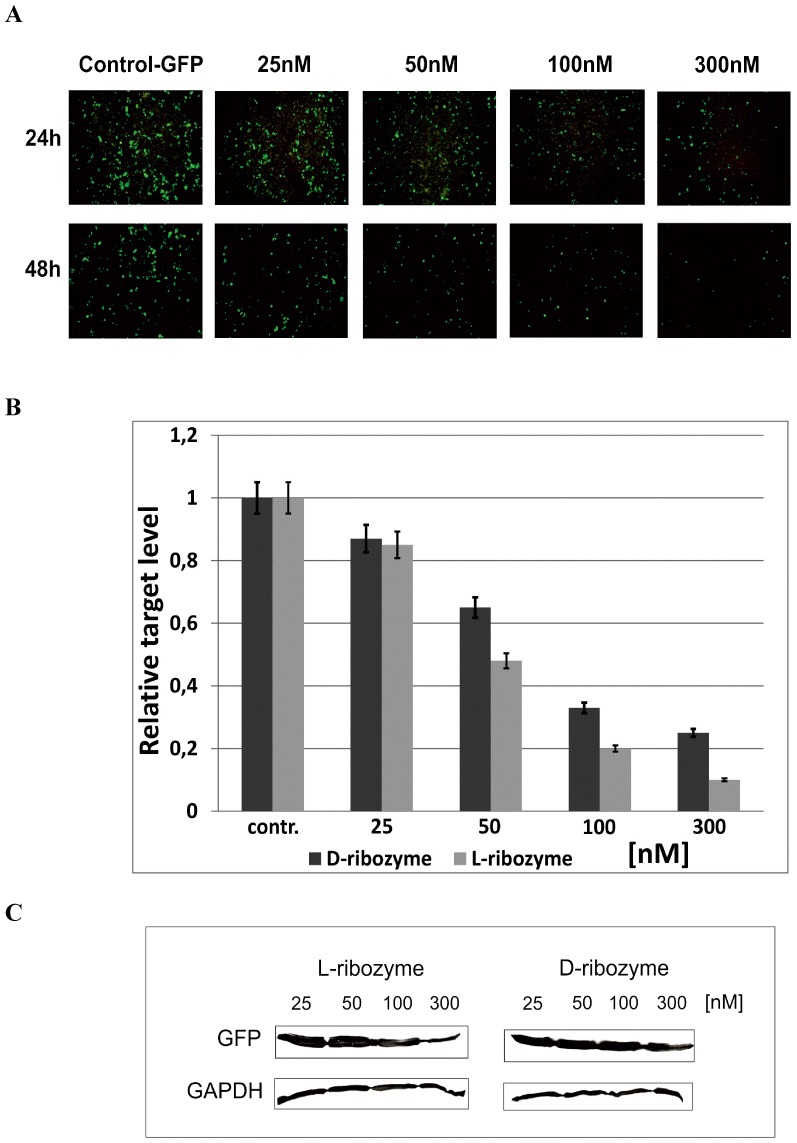
GFP expression in HeLa cells after 24 and 48(L-ribozyme). **(A)** Hydrolysis of GFP mRNAs in HeLA cells after 24 and 48**(B)** Quantification the GFP mRNA hydrolysis by L- and D- hammerhead ribozymes. HeLa cells were transfected with 10, 25, 100, and 300 nM ribozymes and pEGFP. After 48 h of incubation, total RNA was isolated and cDNA was obtained. PCR reactions were performed to assess the GFP expression relative to Hprt and Actb genes. All experiments were repeated at least three times. **(C)** Western blot analysis of proteins from HeLa cells with antibodies against GFP and GAPDH (control).

In order to further substantiate the hydrolysis activities of the L-hammerhead ribozymes with D-RNA targets observed, we transfected HeLa cells with the same hammerhead Spiegelzyme which had previously been used for targeting the chemically synthesized 14-nucleotide-long D-RNA target. The sequence of this target actually corresponded to nucleotides 252 to 265 of the green fluorescent protein (GFP) mRNA. As a check of the efficiency of delivery of L-RNAs into cells, we carried out a transfection of COS-7 cells with the 5′-fluorescein-labelled hammerhead Spiegelzyme in the presence of lipofectamine 2000 (Fig. 6A). Fluorescence within the cells was visible even five days after transfection, which proves that L-RNAs do cross the cell membrane and remain stable in the cytoplasm for longer periods of time. These observations were additionally confirmed by polyacrylamide gel analysis of the L-RNA isolated from COS-7 cells (Fig. 6B). The hammerhead Spiegelzyme was not only stable within the cell, but most importantly, it is catalytically active and reduces the fluorescence due to the green fluorescent protein by hydrolysing its mRNA in HeLa cells (Fig. 7).

The efficiency of GFP mRNA hydrolysis with Spiegelzyme *in vivo* validated with qPCR and Western blot analysis shows that the silencing effect observed on the protein level reached 80% and 60% for hammerhead Spiegelzyme and D-hammerhead ribozyme, respectively (Fig. 7C). The apparently higher activity of the hammerhead Spiegelzyme is most likely due to the fact that it is much more stable in the cells [2] than the D-form of the hammerhead ribozyme in the intracellular environment, where magnesium concentration is less than 1 mM [14].

Thus, the experiments summarized in Figs. 6 and 7 show that the L-hammerhead ribozymes can be introduced into cells by transfection and that the Spiegelzymes are useful tools for controlling gene expression *in vivo*.

Recently, it has been shown that self-replicating L-RNA enzyme built up from nucleotides containing L-ribose can be amplified in a ligand-dependent manner. That experiment showed that amplification of a non-natural molecule as L-RNA is as possible as natural D-RNA [15].

Although the methods of regulation of gene expression using RNA molecules are highly specific, their application as therapeutics is severely limited by the susceptibility of the RNAs to nucleases. In order to stabilize RNA, it has been modified, and the most common modifications are locked nucleic acids (LNA) or substitutions at the 2′-position of the ribose or even its replacement with deoxyribose [16]. Therefore, we were interested to check whether other catalytic nucleic acids, such as the DNAzyme, would be able to form an active heterochiral complex with a D-RNA target. Thus, we designed a mirror-image DNAzyme to test its ability to hydrolyse the 14 nucleotide D-RNA target which we had used in the experiments described above.

**Figure 8:**
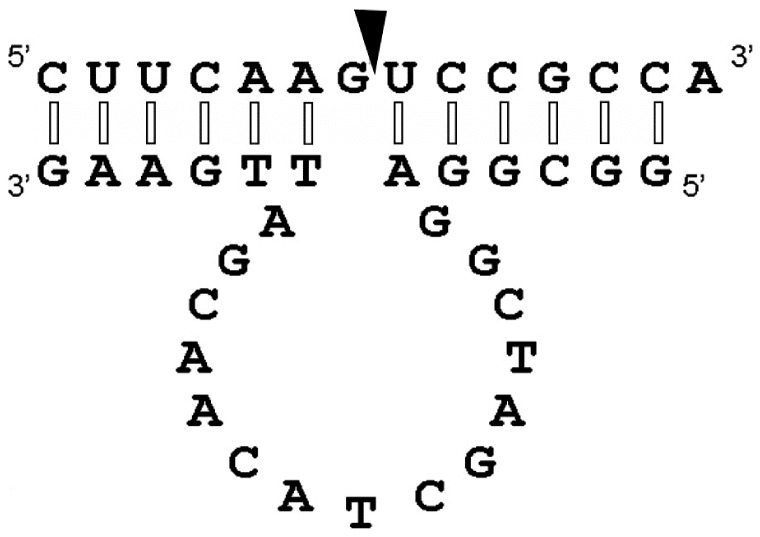
The general model of the secondary structure of D- or L-DNAzyme in the homo- or heterochiral complex with target D-or L-RNA. The arrow shows the anticipated cleavage site. We propose that the interactions between the mirror-image DNAzyme and the D-RNA substrate in the heterochiral complex are Watson-Crick type base pairs in which ribose rings of one strand occur in a rotated orientation (see Figs 12 and also in the Supplementary Figures S1 and S2 and discussion).

The D-DNAzyme designed and its mirror image (Spiegelzyme) were 27 nucleotides long and their potential interactions with the D- and L-RNA substrates of 14 nucleotides length are shown in Fig. 8. In the secondary structural model, the potential cleavage site **G**↓**UC** for a DNAzyme is indicated [17].

**Figure 9:**
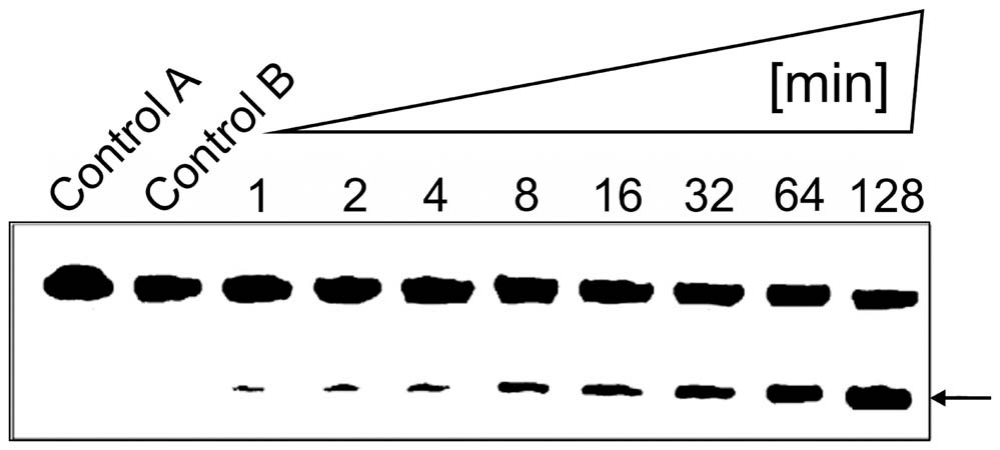
Time-dependent D-RNA hydrolysis by L-DNAzyme. Time-dependent hydrolysis of D-RNA target by L-DNAzyme after 1, 2, 4, 8, 16, 32, 64, and 128 min incubation in Tris-HCl buffer, pH 7.5, 10 mM MgCl_2_, at 37°C. Incubation was performed with 5 µM L-DNAzyme and 0.2 µM target. Control A incubation of D-RNA in Tris-HCl buffer, pH 7.5, without MgCl_2_; Control B with 10 mM MgCl_2_. Both controls were incubated for 128 min. Separation of hydrolysis products was carried out under standard conditions on 20% polyacrylamide gels with 7 M urea. The arrow identifies 7 nt cleavage product.

The results of a time-dependent hydrolysis of the D-RNA target by the mirror-image L-DNAzyme are shown (Fig. 9). Under the conditions chosen, approximately 50% of the D-RNA target was hydrolysed after 128 min (Fig. 9). The L-DNAzyme dependence of the hydrolysis is confirmed by the two Controls A and B, in which the D-RNA target was incubated for 128 min in the absence of the L-DNAzyme in Tris-HCl, pH 7.5, buffer with and without 10 mM MgCl_2_ (Fig. 9).

**Figure 10:**
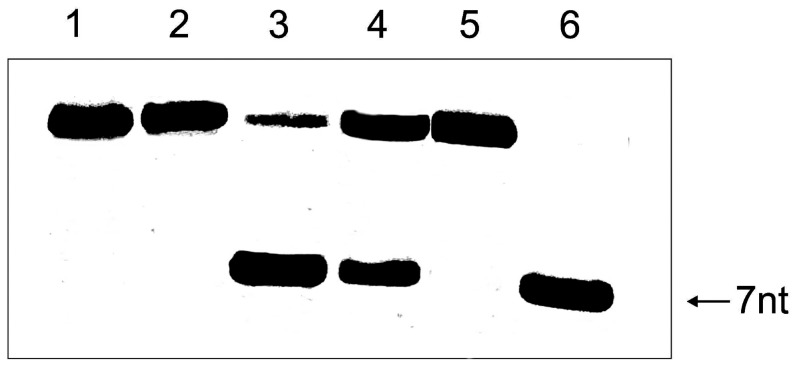
Incubation of D- and L-RNA targets with D-and L-DNAzymes. Lanes 1 and 2: D- and L-RNA targets (0.2 µM) incubated in 50 mM Tris-HCl buffer, pH 7.5, and 10 mM MgCl_2_. Lanes 3 and 4 incubation of D- RNA targets (0.2 µM) with D-and L-DNAzyme (2 µM) in the presence of 10 mM MgCl_2_, lanes 5 and 6 incubation of L-RNA target (0.2 µM) with D-and L-DNAzyme (2 µM) in the presence of 10 mM MgCl_2_. All incubations were carried out for 5 h at 37°C. The arrow shows products of hydrolysis as expected when the RNA target is hydrolyzed by the Spiegelzymes as indicated in Figure 8. Separation of reaction products was accomplished by 20% polyacrylamide gel electrophoresis and 7 M urea.

Using the conditions described above, we next incubated the D- or L-RNA substrates with D- or L-DNAzymes. As can be seen from the results presented in Fig. 10, the D-RNA target was hydrolysed by both the D-DNAzyme and the mirror-image L-DNAzyme, although the L-DNAzyme did not exhibit under these conditions the 100% hydrolysis observed with the D-DNAzyme. Interestingly, when the L-RNA target was incubated with L-DNAzyme or D-DNAzyme, only L-DNAzyme was able to cleave the L-RNA target, while the D-DNAzyme showed no hydrolytic activity towards it.

**Figure 11:**
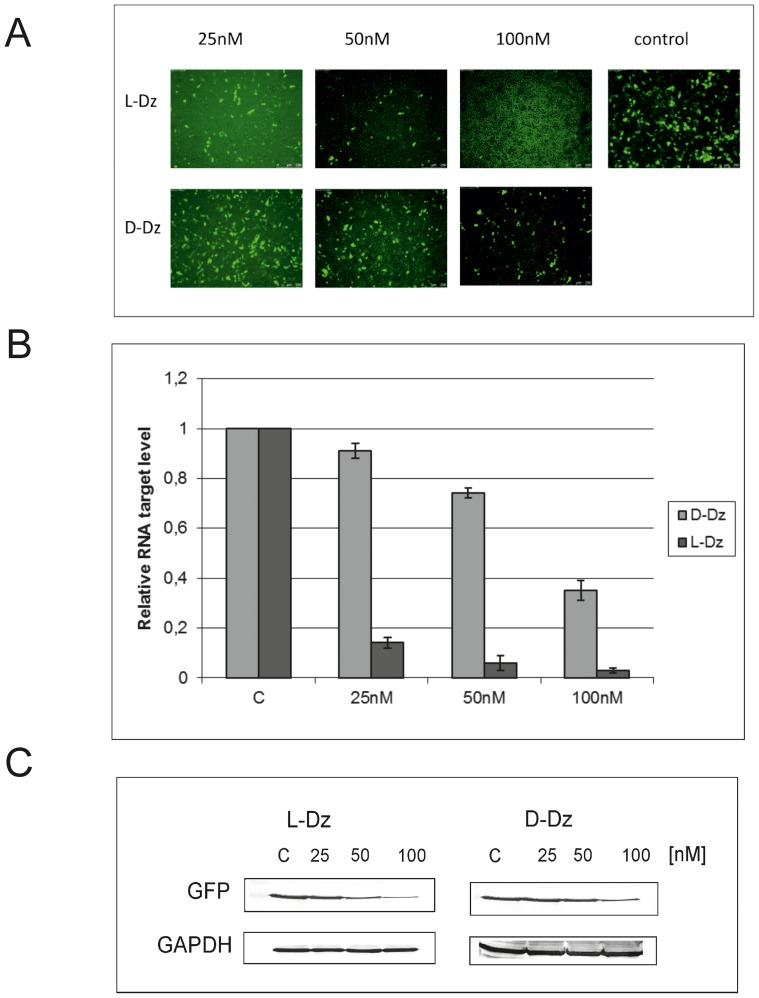
The activity of D-DNAzyme and L-DNAzyme in HeLa cells. **(A)** HeLa cells were transfected with 1 ug EGFP-plasmid and 25, 50 and 100 nM of D-DNAzyme or L-DNAzyme. Fluorescence measurements were carried out 48 h after the transfection. **(B)** Quantification by real-time PCR of the L- and D-DNAzyme hydrolytic activities of GFP mRNAs in HeLa cells. After 48 h, total RNA was isolated and a reverse transcription cDNA was done. PCR reactions were performed to assess GFP expression relative to Hprt and Actb genes in a thermocycler Stratagene Mx3005P instrument. All experiments were repeated at least three times. **(C)** Western blot analysis of GFP gene expression in HeLa cells after transfection with 25, 50 and 100 nM of L-DNAzyme and D-DNAzyme. After 48 h, incubation cells were sonicated in 10 mM Tris HCl, pH 7.5. Proteins were separated and Western blot analysis was carried out.

The results above confirmed our previous observation of an efficient, almost 100% **G**↓**UC**-specific cleavage in homochiral systems of the synthetic D- and L-RNA substrates with D- and L-DNAzymes, respectively [2]. On the other hand, L-RNA is not cleaved with D-DNAzyme (Fig. 10). This observation corresponds well with that of 20 years ago, in which it was shown that L-(dAp)_5_dA formed a duplex with complementary D-poly(rU) [10], [11], [18], but no hybridization could be detected for L-dU oligomer and D-poly(dA) [18], and for complementary L- and D-DNA and RNA containing all four base residues [10], [19]. It is also known that the partial modification of D-DNA with α-L-deoxynucleotides not only increases resistance to nucleases, but also preserves its ability to hybridize with target RNA to induce RNase H cleavage [11]. In order to prove the potential of DNAzyme *in vivo,* we transfected HeLa cells with an L-DNA Spiegelzyme targeted at the G↓UC sequence within the GFP open reading frame. L-DNA Spiegelzyme showed a silencing effect on the GFP expression in HeLa cells, and its activity was slightly higher than that of the corresponding DNAzyme (Fig. 11). It has been shown that DNAzymes intended for use as drugs did not show any adverse side-effects even when applied in relatively high doses in laboratory animals [20]. The mirror-image DNAs also did not induce immunological response like that often observed upon application of other high molecular weight pharmaceuticals.

In this paper, we demonstrated for the first time the construction of Spiegelzymes which can specifically recognise and cleave a target D-RNA at low magnesium concentrations *in vitro* as well as in human serum and in cultured cells. It seems that Spiegelzymes with cleaving capacity for both D- and L-RNA targets might be used in therapeutic and diagnostic applications. Because Spiegelmers [3], [4], like normal aptamers, may have the potential to stimulate unwanted side reactions when used as pharmaceutical reagents, the development of highly specific antidotes would be very desirable, especially if one considers the fact that Spiegelmers are very stable and have a long pharmacological half-life. So far, oligonucleotides have been developed to neutralize unwanted side-effects from aptamers [21]–[24], however, Spiegelzyme catalytic potentials reported in this study would be much better to neutralize the Spiegelmers by sequence-specific hydrolysis and inactivation.

D-hammerhead ribozyme activity against various D-RNAs has been studied for more than 30 years. Crystallographic data of hammerhead ribozymes suggest that the catalytic core has to adopt a well-defined structure that places the nucleophile 2′-oxygen of the target nucleotide for the in-line attack [13].

The biochemical data showing the specificity and activity of the L-hammerhead ribozyme or L-DNAzymes suggest that the catalytic core of both zymes has to adopt the well-defined structure by annealing to the complementary regions of a substrate flanking the cleavage site.

The specific cleavage products of the reaction of D-RNA with Spiegelzymes suggest that interactions between substrate and catalytic molecules are similar or identical to that of D-hammerhead ribozyme or D-DNAzyme.

Formation of such a structure requires base pairs of the sequences flanking the cleavage site of the target D-RNA with the complementary regions of the ribozyme. Since the product obtained with the mirror-image hammerhead ribozyme (Spiegelzyme) is identical to that from the D-hammerhead ribozyme (Fig. 1), it suggests that interactions between the substrate and the catalytic molecules of different chirality will be similar. There is no reference data currently available that would permit an explanation of the mechanism involved concerning the complex formation between D-RNAs and L-ribozymes and the cleavage of the phosphodiester bond by the L-ribozyme. It is interesting that there is no great difference in the outcome of the reactions depending on the chirality of the substrate and ribozyme molecules.

Most of the studies on heterochiral duplexes of nucleic acids in the past were aimed at analysing the L-enantiomer as a possible antisense DNA oligonucleotide. In those studies, there were no attempts to analyse hybridization between all D- and all L-enantiomers of RNA oligonucleotides. The possibility of forming either parallel or anti-parallel heterochiral duplexes has been suggested by NMR studies. Although those results did not provide a definite answer regarding the nature of the interactions, they revealed a strong association between D- and L-RNA penta nucleotides [25].

**Figure 12:**
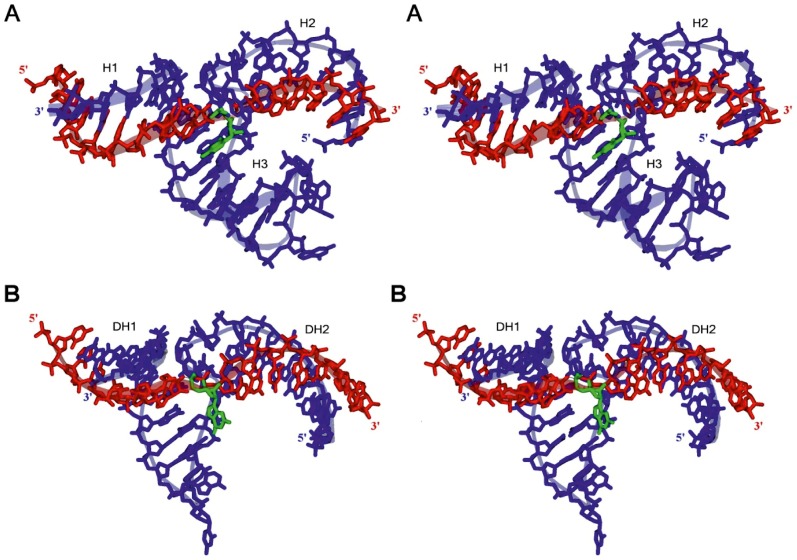
Atomic models proposed for the L-hammerhead ribozyme and the L-DNAzyme interacting with their target D-RNA. **A.** L-HHRz 5′-U**_1_**GGCGCUGAUGAGGCCGAAAGGCCGAAACUUGA**_33_**-3′ (shown in blue) with D-RNA target nucleotide sequence 5′-C**_1_**UUCAAGUCCGCCA**_14_**-3′ (shown in red) with the cleavage site at nucleotide C9 (shown in green). **B**. L-DNAzyme 5′-G**_1_**GCGGAGGCTAGCTACAACGATTGAAG**_27_**-3′ (shown in blue) with same D-RNA target nucleotide sequence 5′-C**_1_**UUCAAGUCCGCCA**_14_**-3′ (shown in red) with the cleavage site at nucleotide G7 (shown in green). See text for detailed discussions of the models.

In order to get a possible insight into understanding the experimental results of the catalytic activity of the heterochiral complexes, we turned to three-dimensional model building. The 3-D models of L-HH-ribozyme and L-DNAzyme with their D-RNA substrates are shown in Fig. 12.

Fig. 12A shows a stereo view of a stick-ribbon model of the L-hammerhead ribozyme in complex with the D-RNA target. It has been created by using the 2.2 Å structure (PDB-ID: 2GOZ) of the full-length catalytically active D-hammerhead ribozyme already published [13].

We can conclude from our 3-D modelling experiments (see Supporting information below) that the sequence-specific cleavage of the D-RNA target by the L-hammerhead ribozyme and the L-DNAzyme is feasible at the cleavage sites observed in our wet experiments.

## Conclusions

With the results presented in this communication we are able to show for the first time that mirror image hammerhead ribozymes and mirror image DNAzymes, which we are calling Spiegelzymes, are capable to cleave in a sequence specific manor natural RNA molecules. We have demonstrated this by specifically cleaving green fluorescent protein mRNAs in HeLa cells. Concerning the specificity, it is of interest that Western Blot experiments show that the mRNA dependent synthesis of several other proteins are not affected by the use of the anti GFP mRNA Spiegelzymes. By extensive molecular modelling experiments we are able to present three-dimensional structural models in which heterochiral interactions between an L-hammerhead ribozyme or an L-DNAzyme with a D-RNA substrate are following Watson-Crick base-pairing rules. The apparent requirement for these interactions is anticlinal conformation of nucleosides**.** The Spiegelzymes described by us recently [2] are potential antidotes against other mirror image nucleic acids to be used in molecular medicine, such as the high affinity mirror image aptamers (Spiegelmers) [3], [4], [6]. The data presented here are enlarging the potential applications of Spiegelzymes into the areas of molecular biology and molecular medicine in which natural occurring RNA molecule sequences are employed, such as mRNAs, microRNAs, siRNAs, etc. Clearly, it will be of great interest to us to explore the use of Spiegelzymes in such directions.

## Supporting Information

Figure S1 **Heterochiral D/L-RNA-duplex showing either right- or left-handedness.** The duplex shown consists of the short sequences D-(5′-CGCCA-3′) and L-(5′-UGGCG-3′) and the base moieties are paired in a canonical Watson-Crick manner. The D-RNA strands are always shown in red, whereas the L-RNA strands are shown in blue. Interestingly, due to the heterochirality of the base pairs, D/L-duplexes can show either right- (**A**) or left-handedness (**B**). **A**. The D/L-RNA duplex shown in its right-handed, anti-parallel conformation. The D-RNA strand (**red**) is in a natural A-RNA form configuration, whereas the nucleotides of the mirror-image L-RNA strand (blue) show ribose rings, which are rotated about the N-glycosidic bonds, which connect the bases with their ribose rings. This arrangement of the nucleotides leads to a smaller minor groove and a larger major groove of the duplex. One can say that the D-RNA strand has forced its natural (right-)handedness upon the L-RNA strand. Only for comparison reasons: The transparent **red** D-RNA strand shown arranged together with the opaque red D-RNA strand would form a natural, right-handed D/D-RNA duplex in A-RNA form. The atom positions of the base moieties of the natural D/D-duplex and that of the D/L-RNA duplex are identical**. B.** The same D/L-RNA duplex, but now shown in its left-handed, anti-parallel conformation. The L-RNA strand (**blue**) is in its preferred left-handed A-form configuration, whereas the nucleotides of the natural D-RNA strand (red) show ribose rings which are rotated about the N-glycosidic bonds connecting their bases. In this case, one can say that the L-RNA strand, for which the natural handedness is left-handed, has forced its handedness upon the D-RNA strand. For comparison reasons: The transparent blue L-RNA-strand shown arranged together with the opaque blue L-RNA strand would form a conventional, left-handed L/L-RNA duplex.
(TIF)

Figure S2 **The detailed view of ribonucleotide conformations in homochiral and heterochiral complexes.** The natural nucleotides are shown in **red**, whereas the mirror-image nucleotides are shown in **blue**: **A.** Comparison between the natural D/D-base pair (above) and the right-handed conformation of the D/L-base pair (below). **B.** Superimposition of the D/D- with the D/L-base pair. **C**. Diagonal side-view of the D/D-base pair superimposed with the D/L-base pair. As one can see, the D-ribose ring and the L-ribose ring show a common symmetry plane as soon as the necessary ribose ring rotation has taken place.
(TIF)

Text S1 **Supporting Information for Proposed Molecular Models.**
(DOCX)
