# Correction: Spiegelzymes® Mirror-Image Hammerhead Ribozymes and Mirror-Image DNAzymes, an Alternative to siRNAs and microRNAs to Cleave mRNAs *In Vivo*?

**DOI:** 10.1371/journal.pone.0093798

**Published:** 2014-03-31

**Authors:** The PLOS ONE Staff

There are errors in Figure S1 and Figure S2. The authors have provided corrected figures here.

## Supporting Information

Figure S1 **Heterochiral D/L-RNA-duplex showing either right- or left-handedness.** The duplex shown consists of the short sequences D-(5′-CGCCA-3′) and L-(5′-UGGCG-3′) and the base moieties are paired in a canonical Watson-Crick manner. The D-RNA strands are always shown in red, whereas the L-RNA strands are shown in blue. Interestingly, due to the heterochirality of the base pairs, D/L-duplexes can show either right- (**A**) or left-handedness (**B**). **A**. The D/L-RNA duplex shown in its right-handed, anti-parallel conformation. The D-RNA strand (**red**) is in a natural A-RNA form configuration, whereas the nucleotides of the mirror-image L-RNA strand (**blue**) show ribose rings, which are rotated about the N-glycosidic bonds, which connect the bases with their ribose rings. This arrangement of the nucleotides leads to a smaller minor groove and a larger major groove of the duplex. One can say that the D-RNA strand has forced its natural (right-)handedness upon the L-RNA strand. Only for comparison reasons: The transparent **red** D-RNA strand shown arranged together with the opaque red D-RNA strand would form a natural, right-handed D/D-RNA duplex in A-RNA form. The atom positions of the base moieties of the natural D/D-duplex and that of the D/L-RNA duplex are identical. **B**. The same D/L-RNA duplex, but now shown in its left-handed, anti-parallel conformation. The L-RNA strand (**blue**) is in its preferred left-handed A-form configuration, whereas the nucleotides of the natural D-RNA strand (red) show ribose rings which are rotated about the N-glycosidic bonds connecting their bases. In this case, one can say that the L-RNA strand, for which the natural handedness is left-handed, has forced its handedness upon the D-RNA strand. For comparison reasons: The transparent blue L-RNA-strand shown arranged together with the opaque blue L-RNA strand would form a conventional, left-handed L/L-RNA duplex.
(TIF)

Figure S2 **The detailed view of ribonucleotide conformations in homochiral and heterochiral complexes.** The natural nucleotides are shown in **red**, whereas the mirror-image nucleotides are shown in **blue**: **A**. Comparison between the natural D/D-base pair (above) and the right-handed conformation of the D/L-base pair (below). **B**. Superimposition of the D/D- with the D/L-base pair. **C**. Diagonal side-view of the D/D-base pair superimposed with the D/L-base pair. As one can see, the D-ribose ring and the L-ribose ring show a common symmetry plane as soon as the necessary ribose ring rotation has taken place.
(TIF)
