# Supplementary material for: Spiegelzymes® Mirror-Image Hammerhead Ribozymes and Mirror-Image DNAzymes, an Alternative to siRNAs and microRNAs to Cleave mRNAs In Vivo?
Source: PLoS One. 2014 Jan 29;9(1):e86673. doi: 10.1371/journal.pone.0086673 (PMC3906056; doi:10.1371/journal.pone.0086673)
Supplement: Text S1 — Supporting Information for Proposed Molecular Models. (DOCX) [file pone.0086673.s003.docx]

**TEXT S1: SUPPORTING INFORMATION FOR PROPOSED MOLECULAR MODELS**

As a first step of the structure of heterochiral complex modelling, a simple copy of the highly conserved catalytic centre of the given molecule (2GOZ) has been used to create the basic structure of the model. All relevant nucleotides of the catalytic centre (G36 as well as A21 in the 2GOZ structure) lie close to each other, to ensure the hydrolysis at the cleavage-site of the substrate (OMC6 in structure 2GOZ and C9 in our model). In the next step, we built up three double helices (H1 to H3 of D-RNA) in a standard A-RNA form using the secondary structure shown in Fig. 1. The shape of the hairpin loop of the double helix H3 has been created automatically by the hairpin loop folding algorithm of the ERNA-3D Molecular Modelling Software (Pentafolium-soft).

After that, we superimposed these three double helices over the three double helices (Stem-I to Stem-III) of the hammerhead ribozyme structure by fitting the bodies of the three helices as well as possible onto the stem helices given. Due to the difference in the nucleotide sequence between the hammerhead ribozyme (structure 2GOZ) and our proposed model, the hammerhead ribozyme has so far been used only as a submittal shape for the spatial path of the nucleotide sequences. The double helices have been superimposed in such a way that the two docking points (the phosphate, respectively, the O3’-atoms) at the ends of each helix, which have to be close enough to both single strands of the catalytic centre, have been used as the starting points of the helices. In this way, we were able to connect the different helices with both single strands of the catalytic centre, without the need to change the spatial shape of the conserved parts of the hammerhead ribozyme structure. After that, the z-component values of the atomic co-ordinates of both RNA strands (the ribozyme as well as the substrate) have been negated. Thus, the mirror image of the whole complex was obtained very easily. The resulting model after this step is a homochiral mirror-image complex of the ribozyme and the RNA-target, in which both strands are in L-configuration and in which all three double helices are left-handed. Subsequently, it was necessary to recreate the D-configuration for the RNA-target strand to get a heterochiral complex. By using the structure creation functions of the ERNA-3D software, we generated a second D-RNA-target single strand with the same original sequence, which had to be superimposed nucleotide by nucleotide onto the L-RNA target strand of the model we had already obtained. Due to the fact that it is impossible to superimpose D-RNA nucleotides and L-RNA nucleotides completely, we overlapped only their flat base moieties onto each other. Nucleic acid bases are flat, because all of their atoms lie on the same plane. Because of their flatness, they are pure two-dimensional units, which also remain congruent if they are mirrored. Their image and their mirror image remain congruent, and therefore, there is no problem to arrange the L- and D-base moieties in such a way that they form canonical Watson-Crick base pairs. In this way, base moieties of the D-RNA can be superimposed exactly on the corresponding bases of the L-RNA target strand of the model already created. The advantage of this approach is that the Watson-Crick base pairs of the resulting heterochiral complex remain completely unchanged, with the plausible propeller twist and with the correct distances for all hydrogen bonds. After this manual superimposition of the bases, one can see that the bonds between the nucleotides of the D-RNA strand were broken, because the distances between the O3’(i)- and the P(i+1)-atoms became too great. This could be easily corrected by the rotation of the D-ribose rings of the nucleotides about their N-glycosidic bonds by reaching a positive anticlinal conformation (+ac).

The delta dihedral angle of these relative rotations lies between -90° and -100° and the resulting dihedral angle χ for the system O4’-C1’---N1-C2 (for pyrimidine bases), respectively O4’-C1’---N9-C4 (for purine bases), is about χ = 99.2° +/- 5° (within the complementary L-RNA strand of the ribozyme, the unchanged dihedral angle for the axes χ is about 160.5° +/- 6°. This angle is positive, because all dihedral angles in mirror-image RNA are negated). From the view of single nucleotides, these relative rotations of the ribose rings correspond to a flipping of the base group into the “inner region” of each nucleotide before the real formation of the base pairing between the D- and L-strands takes place. This inner flipping leads to the most plausible models for heterochiral complexes during the molecular modelling building process. In addition, it is also necessary to adjust the dihedral angles of the backbone atoms slightly between the covalent single bonds between the atoms P, O5’, C5’, and C4’ to get a connected backbone of the D-RNA strand of the duplex. At least, after the D-RNA target strand has been created, fitted and connected, the original L-RNA target strand could be removed from the homochiral model to get the pure heterochiral D/L-RNA complex.

Fig. 12B shows a stereo view of a stick-ribbon model of an atomic model for the L-DNA-zyme in complex with the same D-RNA target and with the cleavage-site at nucleotide G7. This model has been created in the same way as described for our proposed model of the hammerhead ribozyme shown in Fig. 12A, by using the submittal shape of the RNA D-hammerhead ribozyme (PDB-ID: 2GOZ), but by using the secondary structure of Fig. 8. Two hybrid helices (DH1 and DH2), each consisting of one D-RNA and one D-DNA strand, have been created as standard B-form DNA helices. Subsequently, these two helices have been superimposed onto the corresponding stems of the submittal hammerhead ribozyme structure. The docking points (the phosphate, respectively, the O3’-atoms) at the ends of these double helices have been positioned closely enough to the catalytic centre to ensure the simple connection to both single strands of the conserved catalytic centre. In the same way as described in Fig. 12A, we used the spatial shapes of both strands (forming the catalytic centre of the hammerhead ribozyme) to substitute them with the corresponding DNA nucleotides of our proposed DNAzyme model. The nucleotide substitution has been very easily carried out by using the Copy-Structural-Motif function (substitution of nucleotides with nucleotides with different bases, but at the same positions and the same orientations) of the ERNA-3D software. Nucleotides T14 to A18 of the proposed model have also been substituted with the nucleotides of the RNA double helix of the submittal hammerhead ribozyme, but due to the fact that the DNA loop in this case does not form a real double helix, both strands have been pulled away a little bit by using the interactive chain translation method of our ERNA-3D software. In this way, we obtained a shape for the DNA enzymatic loop, which is similar to an RNA double helix, but which does not form canonical Watson-Crick base pairs. Subsequently, a mirror image of this molecule has been created by the negating of the z-components of its atomic co-ordinates, which also leads to an opposite handedness (left-handed) of the participating hybrid double helices. The resulting model after this step is a homochiral mirror-image complex of the DNAzyme and the RNA target. We used exactly the same approach as described for the L-hammerhead ribozyme-D-RNA target complex (see above) to reach the heterochiral L-DNAzyme-D-RNA target hybrid complex.

Due to the lack of crystal structures for both complexes proposed, it is also currently not known how the heterochiral double helices – which are necessary for the composition of these molecules – are really formed in nature. It is known that D-RNA and D-DNA nucleotides form right-handed double helices as soon as they are paired with complementary nucleotides of the same chirality and in complex with other bases which show a canonical base stacking. This behaviour is deeply encoded in the geometrical shape of nucleotides and leads to the conclusion that L-RNA and L-DNA nucleotides tend to form left-handed double helices in the same way. However, due to the heterochirality of the two complexes described, it is also not known what kind of handedness (right- or left-handed) these heterochiral double helices will acquire. Therefore, we constructed structural variants for the D/L-duplex H2 of our proposed L-HHRZ-D-RNA-target complex (see Fig. S1) consisting of the two short RNA strands with the sequences 5’-UGGCG-3’ and 5’-CGCCA-3’ and analysed their structures. The right-handed anti-parallel D/L-RNA duplex conformation has been created by using a right-handed D/D-RNA double helix (in standard A-form) as a submittal shape for canonical base pairs (Fig. S1A).

We used this duplex to superimpose the base groups of the nucleotides of a homonymous L-RNA strand onto the bases of its second D/D-duplex strand, by moving the whole nucleotides. Due to the general flatness of the base groups, their image and their mirror image remain congruent. In this way, base groups of L-RNA nucleotides can be superimposed exactly on the corresponding base groups of the D-RNA strand. This feature is a most important prerequisite for the composition of base pairs within heterochiral double helices. Without the general flatness of these base groups, we assume it would be almost impossible to get “functioning” heterochiral base pairings and the corresponding D/L-duplexes. As soon as the nucleotides of the L-RNA strand have been superimposed onto the base groups of the second D/D-duplex strand, the submittal strand could be removed to get a preliminary model of the D/L-double helix. Interestingly, the superimposition of the base groups did not automatically lead to a connection of the sugar-phosphate backbone of the inserted mirror-image L-RNA strand. Therefore, it is necessary to rotate the L-ribose rings (and the phosphate groups as well) of each nucleotide about their N-glycosidic bonds to end up within a negative anticlinal configuration (-ac). The delta dihedral angles for these relative rotations amount to about 95° with a resulting absolute dihedral angle for the axes χ of about -99.2°. Beyond that, it is necessary to do some smaller adjustments of the dihedral angles within the backbone. The adjusted dihedral angles for the backbone atoms are about: α = O3’-P---O5’-C5’ ~ 140°, β = P-O5’---C5’-C4’ ~ -107°, γ = O5’-C5’---C4’-C3’ ~ -134°, δ = C5’-C4’---C3’-O3’ ~ -84°, ε = C4’-C3’---O3’-P ~ 132°, ζ = C3’-O3’---P-O5’ ~ 47°, which leads to a duplex with a smaller minor groove and a larger major groove compared to a natural A-form of RNA-duplex (Fig. S1, red strands). In this conformation (Fig. S1A), the D-RNA strand (shown in red) of the D/L-duplex has forced its natural right-handedness upon the L-RNA strand (shown in blue). Surprisingly, one and the same D/L-duplex can also show a possible left-handed conformation. Due to the broken molecular symmetry within the heterochiral system, the L-RNA strand of the D/L-duplex is similarly able to force its natural left-handedness upon the D-RNA strand, which leads to the second possible conformation with a left-handed, anti-parallel D/L-duplex shown in Fig. S1A.

In contrast to that, natural D/D-duplexes or the mirror-image L/L-duplexes do not tend to show both left- and right-handedness. In the left-handed duplex, the dihedral angles of the D-RNA strand are identical to the case of the L-RNA strand of Fig. S1, but with an opposite sign. Additionally, the widths of the major and minor grooves are identical to the previous conformation (Fig. S1A). It is important to mention that the occurrence of a sudden switch between either left- or right-handedness (A or B) of the D/L-RNA duplex seems to be very unlikely (or even structurally impossible), because the “decision” for the left- or right-handedness of this complex has to be made before the real composition of the base pairing occurs between the D- and the L-strands. The reason for that can be seen therein, that the necessary base flipping needs space, which is not warranted as long as the bases are jammed within the base stacking of an already composed duplex. Therefore, we assume that this base flipping will only occur while both strands are still separated or even partially separated.

Fig. S2A to Fig. S2C show the single base pair C(D12)-G(L3) of the duplex H2 (see Fig. S2A) of our proposed L-HHRZ-D-RNA-target complex (Fig. S1) as a blow-up. The spatial arrangement of the L-nucleotide is shown within a single (right-handed) heterochiral D/L-base pair compared to the spatial arrangements of the D-nucleotides within a natural Watson-Crick D/D-base pair. As one can see, the atoms of the base moieties are completely congruent (Fig. S2B), and the L- and D-ribose rings of both base pairs show a common symmetry plane (Fig. S2C). In this model, the base group of the L-nucleotide will show an anticlinal conformation (-ac) (angle χ = -99.2°), while the base of the complementary D-nucleotide remains in its natural antiperiplanar configuration (-ap) (angle χ = -166.5°). The anticlinal conformation for the “flipped strand” is, in most cases, valid for A-form D/L-duplexes, because the distance of the rigidly connected atoms O3’(i) and C5’(i+1) of the neighbouring ribose rings (which has to be bridged by the remaining backbone atoms P and O5’) would be a little bit too long within a syn conformation to get a feasible connection between the nucleotides of the strand. The reason is that the distance between the O3’(i) and C5’(i+1) atoms varies depending on the angle χ. However, it would not be valid to say that the anticlinal conformation of the base groups is a prerequisite for such heterochiral D/L-duplexes, because, as an example, the “flipped bases” within a right-handed B-form D/L-duplex will show the angle χ = -85° within a synclinal conformation (-sc), while the complementary strand remains in its anticlinal conformation (-ac) (angle χ = -95°). The overall structure of the duplexes formed is an important factor, therefore, the question whether there will be a preferred syn or anti conformation cannot be easily answered in general.
